# Patient Management Following Percutaneous Coronary Intervention

**DOI:** 10.1016/j.jacadv.2024.101453

**Published:** 2024-12-18

**Authors:** Erica Wennberg, Ali O. Abualsaud, Mark J. Eisenberg

**Affiliations:** a Lady Davis Institute for Medical Research, Jewish General Hospital/McGill University, Montreal, Quebec, Canada; b MD/PhD Program, Temerty Faculty of Medicine, University of Toronto, Toronto, Ontario, Canada; c Institute of Health Policy, Management and Evaluation, Dalla Lana School of Public Health, University of Toronto, Toronto, Ontario, Canada; d Division of Cardiology, Jewish General Hospital/McGill University, Montreal, Quebec, Canada; e Departments of Medicine and of Epidemiology, Biostatistics and Occupational Health, McGill University, Montreal, Quebec, Canada

**Keywords:** coronary artery disease, lifestyle modification, patient management, percutaneous coronary intervention, restenosis, risk factor reduction

## Abstract

Percutaneous coronary intervention (PCI) is a mainstay procedure for the treatment of coronary artery disease. PCI techniques have evolved considerably since the advent of PCI in 1978, and with this evolution in techniques has come changes in the best practices for patient management following PCI. The objective of this review is to provide a comprehensive overview of key considerations in patient management following PCI. The long-term management of patients post-PCI should follow 3 main principles: 1) lifestyle modification and reduction of risk factors; 2) implementation of secondary prevention therapies; and 3) timely detection of restenosis. Best practices in achieving these principles include promotion of smoking cessation, regular physical activity, and a healthy diet, as well as blood pressure, diabetes mellitus, lipid, and weight management; prescription of secondary prevention therapies balancing ischemic and bleeding risk; and avoidance of routine surveillance for restenosis.

## Highlights

• Effective patient management following percutaneous coronary intervention should comprise lifestyle modification and risk factor reduction, implementation of secondary prevention therapies, and timely detection of restenosis.
• Lifestyle modification and cardiac risk factor reduction should include promotion of smoking cessation, regular physical activity, and a healthy diet, as well as blood pressure, diabetes mellitus, lipid, and weight management.
• Medical management of patients should balance ischemic risk with bleeding risk.
• Routine surveillance for restenosis following percutaneous coronary intervention with stress imaging or coronary angiography is generally not recommended.

Percutaneous coronary intervention (PCI) is a mainstay procedure for the treatment of coronary artery disease (CAD). Since the advent of PCI in 1978, PCI techniques have evolved considerably. With this evolution in techniques has come changes in the best practices for patient management following PCI (Table 1). Effective long-term management following successful PCI is essential for the prevention and detection of restenosis and CAD progression. The long-term management of patients post-PCI should follow 3 main principles: 1) lifestyle modification and risk factor reduction; 2) administration of therapies for secondary prevention; and 3) monitoring for timely detection of restenosis (Central Illustration). The goal of this review is to provide a comprehensive overview of key considerations in patient management following PCI, in line with these 3 principles. Key considerations discussed include the timing and components of follow-up assessments, best practices in lifestyle modification and secondary prevention, and rates, timing, clinical presentation of, and risk factors for restenosis. Best practices for restenosis monitoring in view of these factors are also covered, along with choice of functional testing modality for restenosis detection.

**Table 1:** Overview of Recent Developments in and Future Considerations for Patient Management Post-Percutaneous Coronary Intervention

Medical Management	Restenosis Rates	Noninvasive Evaluation
• Guidance on antithrombotic therapy for patients post-PCI has changed in recent years; evidence supports short-term dual antiplatelet therapy in patients with stable CAD and dual antithrombotic therapy for patients with atrial fibrillation. • With advances in medical therapies for CAD, guidance on medical management post-PCI is likely to continue to evolve in coming years.	• PCI technology has continuously improved over the past several decades; restenosis rates have resultingly reduced. • PCI technology is likely to further improve in coming years, resulting in further reductions in restenosis rates.	• With declining restenosis rates over the past several decades, appropriate indications for routine stress testing post-PCI have reduced in number, and it is now considered inappropriate in most cases. • Technological advances have led to improvements in the diagnostic performance of noninvasive testing modalities for restenosis detection over recent years, and new modalities and combinations of modalities are in development. • Further declines in restenosis rates are likely to further reduce appropriate indications for routine stress testing post-PCI in the future. • The diagnostic performance of noninvasive testing modalities is likely to continue to improve over the future, improving system efficiency and patient outcomes.

CAD = coronary artery disease; PCI = percutaneous coronary intervention.

**Central Illustration:**
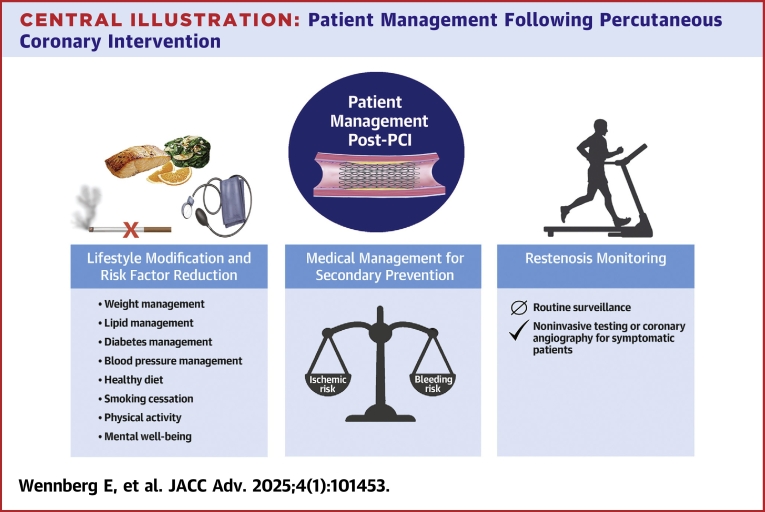
**Patient Management Following Percutaneous Coronary Intervention** The long-term management of patients post-percutaneous coronary intervention (PCI) should follow 3 main principles: 1) lifestyle modification and risk factor reduction; 2) administration of therapies for secondary prevention; and 3) monitoring for timely detection of restenosis.

### Follow-up assessments Post-PCI

Follow-up assessments are usually scheduled at 1 month, 6 months, and 12 months post-PCI. To achieve the 3 aforementioned principles of long-term patient management post-PCI, all follow-up assessments should comprise the following: reinforcement of lifestyle modification, assessment for cardiac risk factor reduction, ensuring participation in exercise rehabilitation, revision of medical therapy, and assessment for recurrent symptoms. Assessment of the access site (radial or femoral) should take place at the first follow-up visit to ensure proper healing and absence of access site complications. For radial access sites, assessment should include visual inspection, hand function assessment, and capillary refill assessment for potential complications (eg, hematoma, compartment syndrome, radial artery occlusion, pseudoaneurysm, nerve injury, infection).^1^ For femoral access sites, assessment should include visual inspection, palpation, and auscultation for potential complications (eg, hematoma, pseudoaneurysm, arteriovenous fistula, infection); if a pseudoaneurysm is suspected, patients should be referred for a duplex ultrasound and vascular surgery consultation.^2^ Follow-up visits can also be taken as an opportunity to ensure patients are up to date on recommended immunizations (eg, influenza, COVID-19, and pneumococcal vaccines).^3^ At the 12-month visit, symptom assessment, risk factor assessment, and medical therapy revision should be undertaken to inform the future care plan. If the patient is asymptomatic at 12 months, their cardiac risk factors are well-controlled, and they are being well-managed medically, follow-up assessment can in most cases be reduced to every 12 months.

### Lifestyle modification and cardiac risk factor reduction Post-PCI

Lifestyle modification and cardiac risk factor reduction post-PCI should include promotion of smoking cessation, regular physical activity, and a healthy diet, as well as blood pressure, diabetes mellitus, lipid, and weight management.^4^ Smoking cessation therapies should be prescribed by cardiologists and initiated in hospital, as opposed to postdischarge at outpatient rehabilitation or smoking cessation clinics (however, referral to these clinics should still be made). Smoking cessation therapies with proven efficacy in patients with cardiovascular disease (CVD) include varenicline, bupropion (stable CVD only), combination pharmacotherapies and nicotine replacement therapies (eg, varenicline + nicotine replacement therapy), individual and telephone counseling, and combination behavioral and pharmacological interventions.^5^, ^6^, ^7^ The 2021 ACC/AHA/SCAI (American College of Cardiology/American Heart Association/Society for Cardiovascular Angiography and Interventions) Guideline for Coronary Artery Revascularization recommends intervention for smoking cessation during hospitalization using a combination of behavioral interventions plus pharmacotherapy, with supportive follow-up for at least 1 month postdischarge.^8^

General recommendations on the timeline to return to regular physical activity post-PCI are after 1 to 2 weeks for patients without myocardial infarction (MI) and after 6 weeks for patients with MI, with heavy lifting and strain on the access site avoided for 2 to 3 days post-PCI. However, there is a lack of evidence-based guidelines in this area. Cardiac rehabilitation can assist with gradual return to exercise. Return to sexual activity can take place several days after PCI provided revascularization was complete and there are no access site complications.^9^ Once return to regular activity is possible, all patients should be counseled to attain a minimum of 150 minutes of moderate-intensity physical activity per week (a minimum of 30 minutes/day for a minimum of 5 days/week) or a minimum of 75 minutes of high-intensity activity per week, if they are physically capable of doing so.^3^^,^^4^^,^^10^ At least 2 days of resistance training per week is additionally recommended.

If clinically indicated, referral to a dietician is advisable to assist with adopting and maintaining a healthy diet. Dietary changes should overall focus on minimization of sodium intake; avoidance of trans fat; reduction and replacement of saturated fat intake with monounsaturated or polyunsaturated fat, complex carbohydrates, and fiber; limiting refined carbohydrates and sweetened beverages; and emphasizing diets composed of vegetables, fruits, legumes, nuts, whole grains, and lean proteins.^3^ European guidelines additionally recommend limiting alcohol consumption.^10^ Patients inquiring about specific dietary plans could be counseled to follow a Mediterranean-style diet.^10^^,^^11^

Blood pressure management should aim for a target of <130/80 mm Hg for patients with hypertension and stable ischemic heart disease.^10^^,^^12^ Patients with hypertension interested in dietary interventions could be counseled to follow the Dietary Approaches to Stop Hypertension diet.^13^ Hemoglobin A1c targets for patients with diabetes mellitus should be individualized. For most patients with CVD, a target of <8% might be appropriate; a target of <7% could also be used if this could be safely attained.^14^ Glucagon-like peptide-1 receptor agonists should be considered for patients with type 2 diabetes; there is strong evidence for their reduction of major adverse cardiovascular events among patients with type 2 diabetes and CVD.^15^ All patients should be initiated on high-dose statins (atorvastatin, rosuvastatin) if tolerated, with a target reduction in low-density lipoprotein cholesterol (LDL-C) of ≥50% versus baseline.^3^^,^^16^ For patients at very high risk (history of multiple major atherosclerotic CVD events or 1 major atherosclerotic CVD event and multiple high-risk conditions), reduction of LDL-C to <55 mg/dL should additionally be targeted.^17^ European guidelines also recommend high-dose statins for all patients, with a target of <55 mg/dL and ≥50% reduction for all patients with chronic coronary syndrome.^10^ If LDL-C targets are not achieved with single high-dose statins, ezetimibe can be added.^3^^,^^10^^,^^17^ If targets are still not achieved, PCSK9 monoclonal antibodies can be considered. Patients with continued difficulty in achieving targets could be referred to a lipid specialist.

If weight management cannot be achieved with lifestyle interventions, pharmacological therapy could be considered. Emerging evidence on glucagon-like peptide-1 receptor agonists suggests they could hold a future role in secondary prevention of CVD. The SELECT (Semaglutide Effects on Cardiovascular Outcomes in People with Overweight or Obesity) trial found that weight loss with semaglutide therapy resulted in a reduction in major adverse cardiovascular events compared to placebo among patients with preexisting CVD and overweight or obesity but no history of diabetes.^18^ Given the role of inflammation in atherosclerosis, low-dose colchicine therapy has been proposed for secondary prevention of CVD^3^^,^^19^ and could be considered for patients post-PCI. The COLCOT (Colchicine Cardiovascular Outcomes Trial) trial randomized 4,745 patients within 30 days of an MI treated with PCI to daily low-dose colchicine or placebo and found that colchicine reduced the risk of ischemic cardiovascular events.^19^ However, the colchicine group had a significantly higher incidence of pneumonia.

The 2021 ACC/AHA/SCAI Guideline for Coronary Artery Revascularization recommends prescription of a comprehensive cardiac rehabilitation program post-PCI to reduce patient mortality and hospital readmissions and improve quality of life.^8^ Participation in an exercise-based cardiac rehabilitation program post-PCI is also recommended by European guidelines.^10^ Prescription can take place either before hospital discharge or during the first outpatient visit, and the program can be home-based or center-based. Cardiac rehabilitation programs combine medical evaluation, risk factor modification, patient education, counseling, and exercise training.^20^ Both home-based and center-based programs have been shown to provide a constellation of benefits including reduced mortality and cardiac events as well as improved quality of life, exercise tolerance, lipid levels, blood pressure, and smoking cessation.^21^, ^22^, ^23^

The mental well-being of patients should be monitored post-PCI. Following PCI, patients may experience depression, anxiety, and stress, which, in addition to burdening patients psychosocially, can reduce adherence to treatment and risk factor management and increase the risk of recurrent cardiac events.^24^ In patients with symptoms indicative of depression, anxiety, or stress post-PCI, psychological evaluation and intervention should be initiated. The 2021 ACC/AHA/SCAI Guideline for Coronary Artery Revascularization recommends treatment of these patients with cognitive behavioral therapy, psychological counseling, and/or pharmacological interventions to improve quality of life and cardiac outcomes.^8^

### Medical management Post-PCI

Medical therapy reassessment should be performed post-PCI to ensure that patients are receiving appropriate medical therapy for secondary prevention. In completely revascularized patients, antianginal medications (eg, beta-blockers, nitrates, calcium channel blockers) can often be discontinued, provided they are not needed for other indications. The 2021 ACC/AHA/SCAI Guideline for Coronary Artery Revascularization recommends against beta-blocker use for secondary prevention post-PCI in completely revascularized patients with stable ischemic heart disease and normal left ventricular function.^8^ In patients with comorbid conditions (eg, hypertension, atrial fibrillation), appropriate medications for treatment of these conditions should be continued. Given historical associations between menopausal hormone therapy and thrombosis, female, postmenopausal patients might request counseling on its safety post-PCI. With newer agents and transition toward lower doses, shorter duration, and earlier onset of therapy, contemporary evidence suggests venous thrombosis as the main cardiovascular concern related to hormone therapy.^25^ However, the ACC CVD in Women Committee recommends that systemic hormone therapy be generally avoided in women with atherosclerotic CVD and that alternative therapy be considered.^26^ Low-dose vaginal estrogen therapy is considered safe for all patients, and individualized, shared decision-making can be considered for those with persistent, severe symptoms.

When determining type and duration of antiplatelet therapy post-PCI, secondary prevention of ischemic events should be balanced with bleeding risk. Recommendations for achieving this differ between patients with stable CAD and acute coronary syndrome (ACS). Among patients with stable CAD, recent evidence supports prescription of short duration (1-3 months) dual antiplatelet therapy (DAPT; aspirin plus a P2Y12 inhibitor, eg, clopidogrel, ticagrelor, or prasugrel) followed by single antiplatelet therapy (SAPT). In patients treated with second-generation drug-eluting stents (DES), a meta-analysis of 5 randomized controlled trials found that short duration (1-3 months) DAPT followed by P2Y12 inhibitor SAPT led to reduced bleeding events at 12 months post-PCI compared to 12 months of DAPT.^27^ There was no difference in stent thrombosis, all-cause death, MI, and stroke. An exploratory analysis showed short-duration DAPT followed by aspirin SAPT produced similar results. The 2021 ACC/AHA/SCAI Guideline for Coronary Artery Revascularization recommends that short duration DAPT (1-3 months) with subsequent transition to P2Y12 inhibitor monotherapy is reasonable in select patients post-PCI^8^; recommendations from the 2024 ESC (European Society of Cardiology) Guidelines for the management of chronic coronary syndromes are similar.^10^

Among patients with ACS, DAPT should be continued for 12 months in the absence of complications, with continued aspirin SAPT thereafter.^8^ However, there is evidence supporting DAPT de-escalation strategies (transition from a more potent to a less potent P2Y12 inhibitor) and short duration DAPT. An individual patient meta-analysis of 4 randomized controlled trials in patients with ACS treated with PCI (n = 10,333) found that DAPT with P2Y12 inhibitor de-escalation was associated with reduced ischemic (cardiac death, MI, and cerebrovascular events) and bleeding events at 12 months post-PCI compared to standard DAPT.^28^ The TWILIGHT (Ticagrelor with Aspirin or Alone in High-Risk Patients after Coronary Intervention) trial randomized 7,116 high-risk patients with ACS after 3 event-free months on DAPT post-PCI to ticagrelor alone and ticagrelor plus aspirin and found reduced risk of bleeding in the ticagrelor group with no difference in death, MI, or stroke.^29^ Prolonged DAPT beyond 12 months can be considered for patients who are not at high risk of bleeding and have not experienced a bleeding complication on DAPT.^8^ High thrombotic risk (eg, left main stents, extensive use of stents, bifurcation stents, and repeat stenting following in-stent thrombosis) could be an appropriate indication. Low-dose colchicine therapy has recently been proposed as an alternative to aspirin therapy post-PCI to reduce bleeding risk. While evidence for its safety and effectiveness for this purpose is limited, a recent pilot trial demonstrated feasibility of administering low-dose colchicine plus ticagrelor or prasugrel therapy at day 1 post-PCI with discontinuation of aspirin among patients with ACS treated with DES.^30^

For both patients with stable CAD and ACS, situations in which early discontinuation of DAPT could be appropriate include those who require surgery, who experience a bleed (eg, gastrointestinal or genitourinary) on DAPT, or who are at high bleeding risk. Patient age is an important consideration in assessment of bleeding risk. For older and high-risk patients on DAPT or P2Y12 inhibitor monotherapy for whom gastrointestinal bleeding is a concern, proton pump inhibitors should be prescribed concomitantly for gastroprotection.^10^^,^^31^^,^^32^ There is some concern that proton pump inhibitors (specifically omeprazole) could reduce the effectiveness of clopidogrel; however, there is no definitive evidence that this interaction is clinically meaningful.^31^^,^^32^ Choice of DAPT agents and dosages should also balance bleeding and ischemic risk^33^; less potent agents and lower dosages should be considered among older patients and other patients at high bleeding risk. Potential drug-drug interactions should also be considered in the choice of P2Y12 inhibitor. Overall, there are more potential interactions described for clopidogrel and ticagrelor than for prasugrel, and drugs that are strong CYP3A4 inducers or inhibitors should generally be avoided.^34^

**Figure 1:**
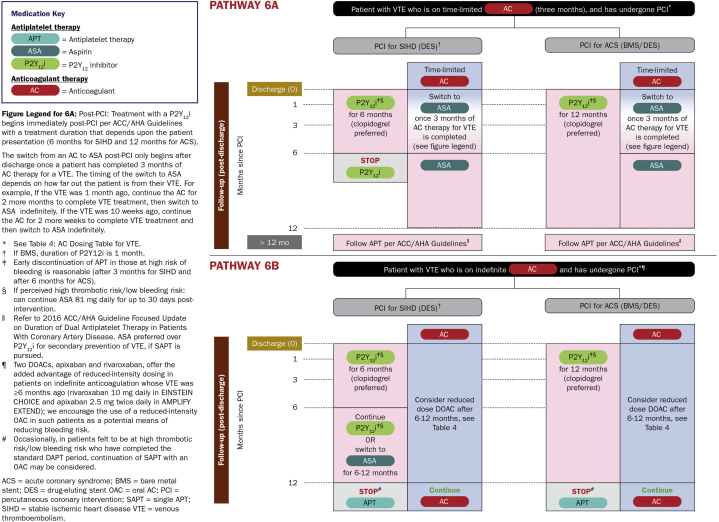
**2020 ACC Expert Decision Pathway for a Patient With Venous Thromboembolism on Time-Limited or Indefinite Anticoagulation Therapy and Who Has Undergone Percutaneous Coronary Intervention**^39^ Reprinted with permission from Kumbhani DJ, Cannon CP, Beavers CJ, et al, 2020 ACC expert consensus decision pathway for anticoagulant and antiplatelet therapy in patients with atrial fibrillation or venous thromboembolism undergoing percutaneous coronary intervention or with atherosclerotic cardiovascular disease: A report of the American college of cardiology solution set oversight committee. J Am Coll Cardiol. 2021;77(5):629-658. https://doi.org/10.1016/j.jacc.2020.09.011.

For patients taking anticoagulant therapy (eg, patients with atrial fibrillation, with mechanical prosthetic valves, and with prior venous thromboembolism), triple antithrombotic therapy (DAPT plus an oral anticoagulant) was formerly the mainstay medical therapy post-PCI. Evidence now supports the use of dual antithrombotic therapy (SAPT plus an oral anticoagulant) in these patients to mitigate bleeding risk. A network meta-analysis of randomized controlled trials of patients with atrial fibrillation found that direct oral anticoagulant (DOAC) and P2Y12 inhibitor therapy post-PCI was associated with reduced bleeding risk compared to vitamin K antagonist plus DAPT, without a significant difference in major adverse cardiovascular events.^35^ The 2021 ACC/AHA/SCAI Guideline for Coronary Artery Revascularization recommends discontinuation of aspirin treatment 1 to 4 weeks post-PCI with maintenance of P2Y12 inhibitor plus DOAC or warfarin therapy to reduce bleeding risk in patients with atrial fibrillation taking oral anticoagulant therapy.^8^ Duration of triple antithrombotic therapy should be limited to 1 week post-PCI for most patients, with duration up to 4 weeks considered for patients at high thrombotic and low bleeding risk.^36^ The 2024 ESC Guidelines for the management of chronic coronary syndromes recommend early (≤1 week) cessation of aspirin therapy after uncomplicated PCI, with continuation of oral anticoagulant and clopidogrel therapy thereafter.^10^ The HAS-BLED score can be used to guide decision-making regarding anticoagulation therapy and bleeding risk in patients with atrial fibrillation.^37^ The 2021 ACC/AHA/SCAI guideline additionally recommends that choice of a DOAC over warfarin is reasonable to reduce bleeding risk in patients with atrial fibrillation taking oral anticoagulant therapy and DAPT or P2Y12 inhibitor monotherapy.^8^ Dual antithrombotic therapy should be continued for up to 12 months post-PCI with transition to oral anticoagulant monotherapy thereafter; early discontinuation at 6 months can be considered for patients with low thrombotic or high bleeding risk.^10^^,^^36^ Based on a secondary analysis of the AFIRE (Atrial Fibrillation and Ischemic Events with Rivaroxaban in Patients with Stable Coronary Artery Disease) trial, monotherapy with rivaroxaban could be considered for patients with atrial fibrillation and stable CAD beyond 12 months post-PCI.^38^ For patients post-PCI with prior venous thromboembolism on indefinite anticoagulation, a DOAC should be combined with SAPT (Figure 1).^39^ Patients with mechanical valves must receive warfarin in combination with SAPT post-PCI, as DOACs are contraindicated in these patients.^40^

### Restenosis

#### Rates and timing of restenosis

**Figure 2:**
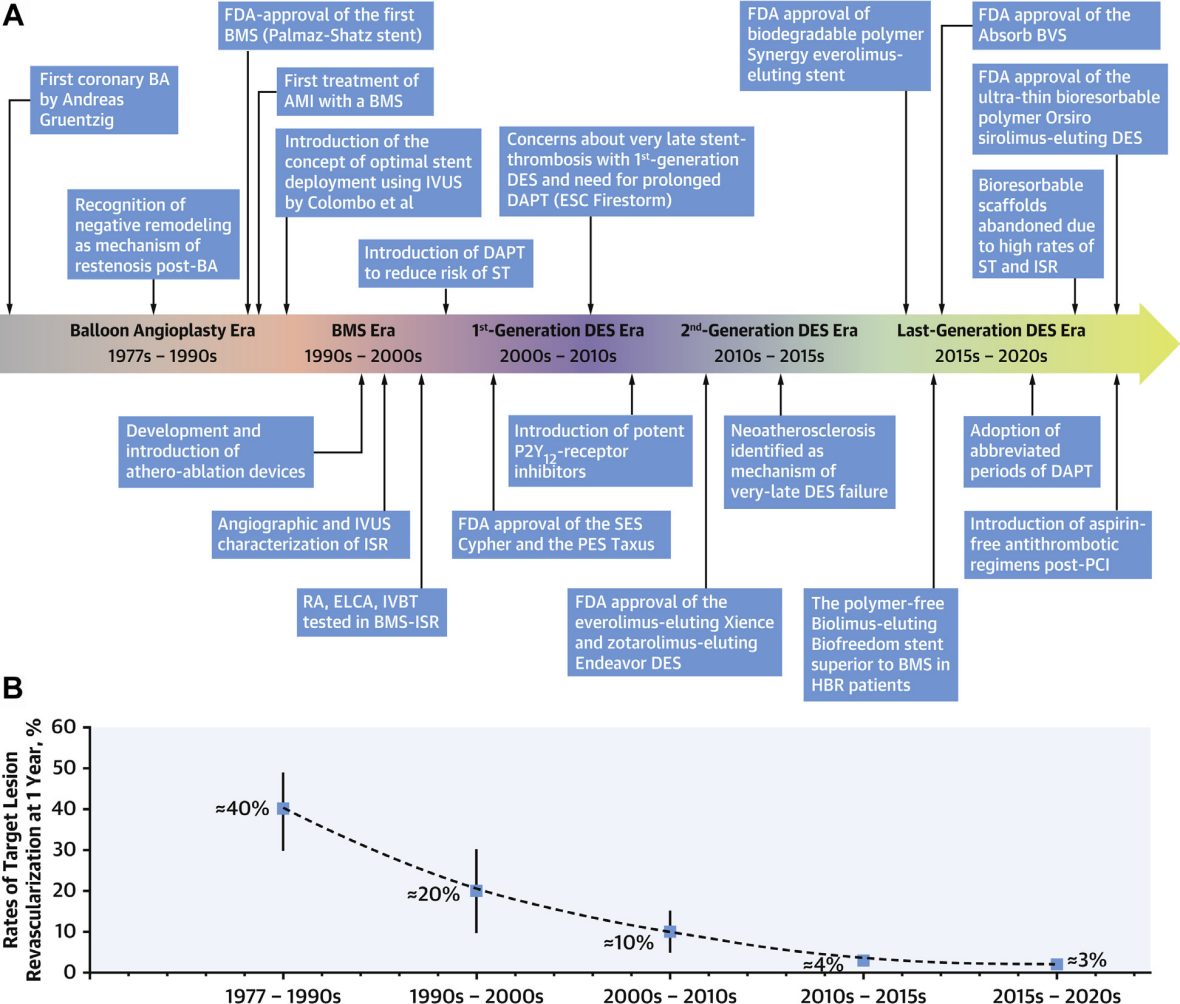
**Advances in Percutaneous Coronary Intervention Techniques and Corresponding Reductions in Restenosis Rates**^41^ (A) Evolution in DES technologies, PCI techniques, and pharmacotherapies over the last 4 decades, (B) paralleled by a substantial reduction in the rates of TLR. Reprinted with permission from Giustino et al.^41^

Restenosis rates post-PCI have decreased steadily since the advent of PCI in 1978 as a result of advances in stent technology. In the era of balloon angioplasty, restenosis rates (as measured by target lesion revascularization) of up to 40% at 1 year were observed (Figure 2).^41^ With the introduction of bare-metal stents (BMS), restenosis rates were decreased to ∼20%; with the subsequent introduction of first-generation DES, restenosis rates were further decreased to ∼10%. Restenosis rates continued to decline with advances in DES technology to <5% with second-generation DES. The typical time course for restenosis with BMS implantation is within 12 months post-PCI; with DES implantation, the time course for restenosis may exceed 12 months.^41^ A meta-analysis of 19 randomized trials found rates of ischemia-driven target lesion revascularization were 14.7% for BMS and 2.5% for second-generation DES within 1 year post-PCI.^42^ From 1 to 5 years post-PCI, rates of ischemia-driven target lesion revascularization were 6.1% for BMS and 4.4% for new-generation DES.

#### Risk factors for restenosis

Risk factors for restenosis post-PCI fall into 3 major categories: clinical, anatomical, and procedural. Clinical risk factors include diabetes mellitus, chronic kidney disease, older age, hypertension, hypersensitivity reactions, and history of bypass surgery.^43^, ^44^, ^45^ Anatomical risk factors include long lesions, small vessel diameters, and chronic total occlusion.^43^, ^44^, ^45^ Procedural risk factors include balloon angioplasty alone or BMS implantation (vs DES), first- versus second-generation DES implantation, long stents, and suboptimal stent deployment or placement (eg, under expansion, overlapping, stent gap, geographic miss).^43^, ^44^, ^45^

#### Clinical assessment and presentation of restenosis

Clinical assessment for restenosis should be guided by 3 main factors: patients’ pretest likelihood of restenosis development, their likelihood of symptom development in the event of restenosis, and their likelihood of experiencing an adverse event in the event of restenosis. These likelihoods can vary as a function of both patient-level (eg, comorbidities) and stent-level factors (eg, stent type and placement). Restenosis most often presents as ACS, with unstable angina being most common and MI less common, followed by stable angina and silent ischemia.^46^^,^^47^ Importantly, a large proportion of patients with angiographic restenosis may not have anginal symptoms. Historically, rates of asymptomatic restenosis were estimated to be ∼50%.^48^ However, this estimate was based on studies of patients treated with balloon angioplasty as well as stented patients, and rates might have declined since; a more recent cohort study of 10,004 patients stented with BMS and DES found 36% of patients with angiographic restenosis at 6 to 8 months were asymptomatic.^49^ Among stented patients, the severity of restenosis presentation has been found to depend largely on patient-level as opposed to stent-level factors, with higher comorbidity (eg, hypertension, current use of cigarettes, chronic renal failure) generally associated with more severe presentation.^46^ More severe presentation (ie, ACS) is in turn associated with greater risk of experiencing adverse cardiovascular events. Treatments for restenosis include repeat stenting, drug-coated balloons, vascular brachytherapy, atherectomy, and coronary artery bypass grafting and are described further elsewhere. For first-episode clinical in-stent restenosis, repeat stenting with a DES is generally recommended.^8^ For recurrent in-stent restenosis, coronary artery bypass grafting could be considered. Nonclinical restenosis can often be treated medically.

#### Invasive evaluation for restenosis: coronary angiography

Coronary angiography is the gold standard for diagnosis of restenosis. However, routine surveillance of patients post-PCI with coronary angiography is not appropriate. Coronary angiography is advisable in patients with recurrence of pre-PCI symptoms or who have noninvasive findings suggestive of restenosis. In select situations, such as recurrence of prior cardiac symptoms, coronary angiography can be used as a first-line diagnostic test for restenosis; however, it is in most cases advisable to defer coronary angiography pending the results of noninvasive testing. Per the current ACCF/SCAI/AHA Appropriate Use Criteria for Diagnostic Catheterization, diagnostic catheterization is appropriate in patients post-PCI with intermediate- or high-risk noninvasive findings and worsening or limiting symptoms, and it is inappropriate in those who are asymptomatic or have stable symptoms.^50^ The multisociety 2023 Multimodality Appropriate Use Criteria for the Detection and Risk Assessment of Chronic Coronary Disease (2023 Appropriate Use Criteria for CCD) consider cardiac catheterization appropriate in symptomatic patients post-PCI with symptoms similar to their prior ischemic episode and/or anginal symptoms.^51^ Routine coronary angiography post-PCI is likely to increase system burden without improvement in clinical outcomes. A randomized trial found routine follow-up coronary angiography at 1 year post-PCI resulted in increased repeat revascularization without clinical benefit.^52^

#### Noninvasive evaluation for restenosis

Noninvasive testing is frequently used to evaluate patients following PCI. Most noninvasive tests are stress testing modalities, which comprise elicitation of myocardial ischemia through exercise or pharmacological stress with concurrent monitoring. The diagnostic performance of noninvasive evaluation modalities is reliant on patients’ pretest probability of restenosis and the test modality used. Compared with invasive evaluation (coronary angiography), noninvasive testing presents a time, safety, and cost advantage. It can be used following PCI for indications unrelated to symptom presentation, such as patient reassurance, exercise prescription, entrance to a cardiac rehabilitation program, and clearance to operate heavy machinery or to pilot airplanes. Examples of noninvasive testing include exercise electrocardiography (ECG), stress radionuclide imaging (RNI), stress echocardiography, coronary computed tomography angiography (CCTA), and stress cardiac magnetic resonance (CMR); specific considerations for use of these modalities post-PCI are explored in greater detail in subsequent sections. Whether or not to perform noninvasive testing for restenosis in a given patient, and which modality to use, should take into account the patient’s symptom presentation and the goal of noninvasive testing. In most cases (except in patients at high risk from restenosis and patients needing clearance to return to work), noninvasive testing of asymptomatic patients is considered inappropriate.

### Noninvasive testing modalities

#### Exercise electrocardiography

Exercise ECG is the most frequently employed evaluation method for patients post-PCI. When used alone, exercise ECG performs poorly in diagnosing restenosis. However, it can provide clinically useful information regarding symptom burden and functional capacity, notably prior to initiation of cardiac rehabilitation or exercise programs. In a meta-analysis of patients who underwent exercise ECG alone >6 months after successful PCI, the pooled sensitivity and specificity of exercise ECG for the identification of restenosis was 46% and 77% with restenosis defined as coronary artery stenosis ≥50%.^53^ The multisociety 2023 Appropriate Use Criteria for CCD consider ECG treadmill testing appropriate in symptomatic patients prior to cardiac rehabilitation with no new or worsening symptoms; it is the only modality with an appropriate rating in this category.^51^ It is considered potentially appropriate in symptomatic patients post-PCI with incomplete revascularization, symptoms similar to their prior ischemic episode and/or anginal symptoms, or nonanginal symptoms.

#### Imaging-based modalities

Imaging-based modalities are in general superior to exercise ECG for the detection of restenosis with regard to diagnostic performance.^53^ The multisociety 2023 Appropriate Use Criteria for CCD consider stress RNI (myocardial perfusion imaging), stress echocardiography, and stress CMR appropriate in symptomatic patients post-PCI with incomplete revascularization or with symptoms similar to their prior ischemic episode and/or anginal symptoms, and as potentially appropriate in patients with nonanginal symptoms.^51^ The 2018 ESC/EACTS (European Association for Cardio-Thoracic Surgery) Guidelines for myocardial revascularization recommend imaging stress tests over stress ECG for symptomatic patients.^54^ Existing guidelines do not recommend use of a particular stress RNI modality over another in patients post-PCI. However, positron emission tomography myocardial perfusion imaging (MPI) with rubidium-82 may offer several advantages compared to conventional single photon emission computed tomography MPI, including higher diagnostic accuracy, shorter procedure length, decreased radiation exposure, and higher image quality.^55^^,^^56^ In a study of 236 patients post-PCI, stress CMR with first-pass perfusion and late gadolinium enhancement had a sensitivity of 91% and a specificity of 90% for detection of ≥70% restenosis.^57^ Due to the ferromagnetic properties of some stents, there is some concern of stent migration or heating being induced by CMR; however, its use appears safe in stented patients.^58^

Other imaging-based modalities with proven value in CAD evaluation (eg, CCTA) have less evidence for value in restenosis detection. The multisociety 2023 Appropriate Use Criteria for CCD consider CCTA rarely appropriate in symptomatic patients post-PCI with incomplete revascularization, and potentially appropriate in patients with nonanginal symptoms or with symptoms similar to their prior ischemic episode and/or anginal symptoms.^51^ The 2021 SCCT Expert Consensus Document on CCTA^59^ and the 2010 multisociety Appropriate Use Criteria for Cardiac Computed Tomography^60^ recommend that CCTA is only appropriate for restenosis assessment of left main stents ≥3 mm in diameter. The limitation to stents of larger size is due to CT artifacts (blooming, beam hardening, motion) associated with stent metals. Techniques combining the anatomical assessment of CCTA with physiologic assessments, including fractional flow reserve (FFR) CT and static and dynamic CT MPI, have evolved in recent years. In a study of 33 stented patients, machine learning FFR CT was shown to be feasible for restenosis detection and demonstrated prognostic value for adverse event prediction in patients at low-moderate risk.^61^ In a study of 150 stented patients, concordant CCTA and CT MPI had a significantly improved diagnostic rate and accuracy for restenosis detection over CCTA alone, compared to a reference standard of coronary angiography with FFR.^62^

#### Routine stress testing

With the advent of DES and resultant reductions in restenosis rates, appropriate indications for routine stress testing post-PCI have decreased. Use of routine stress testing can lead to high rates of false positive tests, increased system burden, and unnecessary invasive evaluation without associated clinical benefit. Appropriate indications for stress testing of asymptomatic patients post-PCI are at present typically limited to specific high-risk patient subgroups or for purposes of exercise program or cardiac rehabilitation assessment. The multisociety 2023 Appropriate Use Criteria for CCD consider stress testing rarely appropriate in asymptomatic patients <2 years post-PCI and potentially appropriate in asymptomatic patients >2 years post-PCI, with prior high-risk PCI, or with incomplete revascularization.^51^ By modality, stress testing with stress nuclear MPI, stress echocardiography, or stress CMR is only considered appropriate in asymptomatic patients with a history of or at high risk for silent ischemia. CCTA and treadmill ECG are considered potentially appropriate in these patients. Exercise ECG is considered appropriate in asymptomatic patients prior to cardiac rehabilitation or in patients with known chronic coronary disease prior to initiating an unsupervised exercise program.

**Figure 3:**
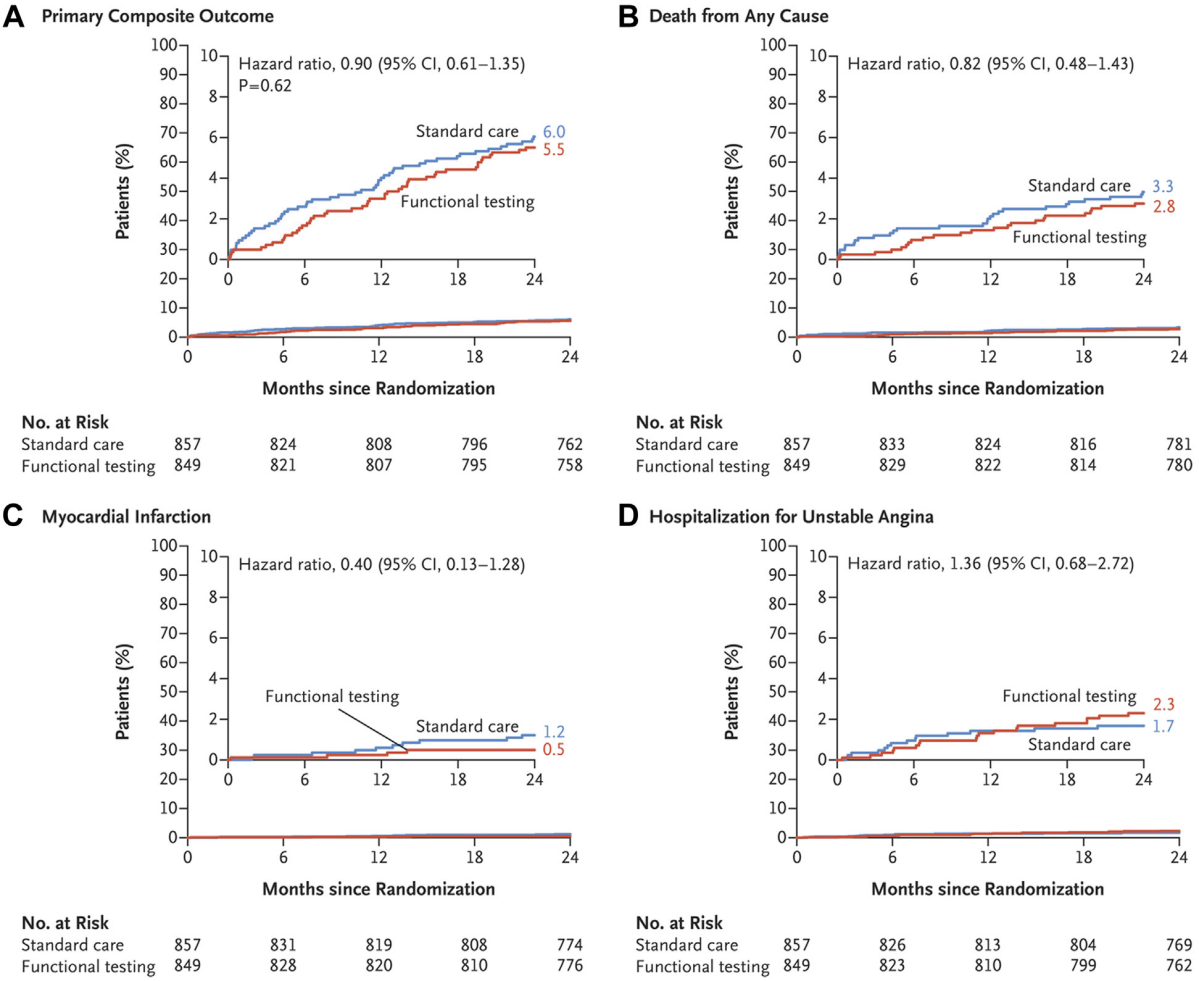
**Findings of the POST-PCI Trial**^63^ (A) Cumulative incidence of the primary composite outcome of death from any cause, myocardial infarction, or hospitalization for unstable angina. (B) Cumulative incidence of death from any cause; (C) cumulative incidence of myocardial infarction. (D) Cumulative incidence of hospitalization for unstable angina. Reprinted with permission from Park et al.^63^

**Figure 4:**
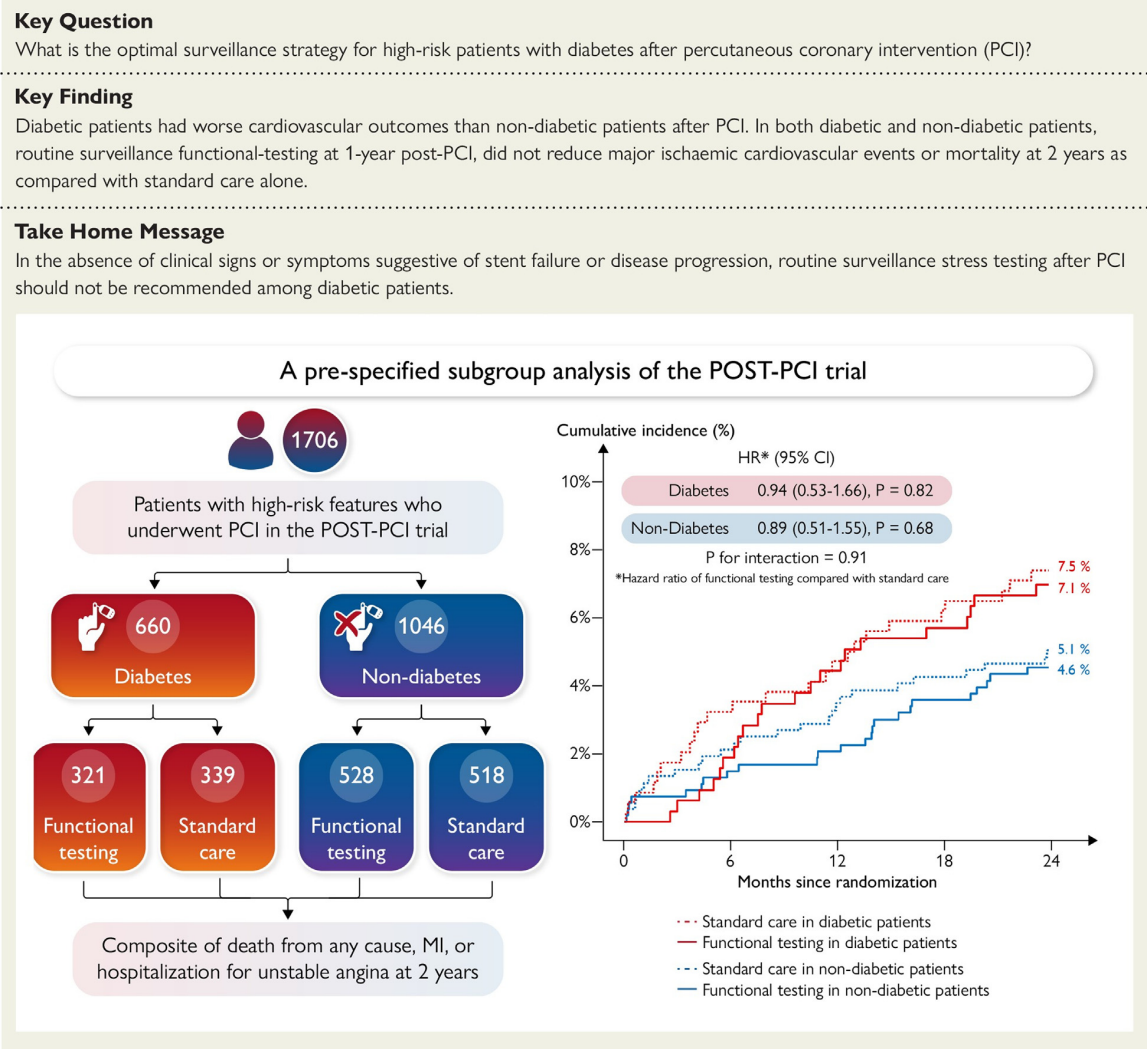
**Findings of the POST-PCI Trial Subgroup Analysis of Patients With Diabetes**^64^ Reprinted with permission from Kim et al.^64^

The utility of routine stress testing for restenosis following PCI has been examined in several major clinical trials using a variety of short- and long-term clinical outcomes. The POST-PCI (Pragmatic Trial Comparing Symptom-Oriented versus Routine Stress Testing in High-Risk Patients Undergoing Percutaneous Coronary Intervention) trial randomized 1706 patients with high-risk anatomical or clinical characteristics undergoing PCI to routine functional testing (nuclear stress testing, exercise ECG, or stress echocardiography) at 1 year compared to standard care alone, which included stress testing for clinical indications.^63^ At 2 years post-PCI, the trial found routine testing led to an increase in invasive coronary angiography and repeat revascularization with no difference in major adverse cardiac events (MI and hospitalization for unstable angina) or mortality (Figure 3). Prespecified subgroup analyses of the POST-PCI trial found no benefit of routine testing among 660 patients with diabetes who underwent high-risk PCI (Figure 4)^64^ or among 526 patients with ACS who underwent high-risk PCI.^65^ In the ADORE (Aggressive Diagnosis of Restenosis)^66^ and ADORE II^67^ trials, 348 PCI patients and 84 high-risk PCI patients, respectively, were randomized to routine exercise stress testing at 6 weeks and 6 months post-PCI compared to selective use of exercise stress testing for clinical indications. At 9 months post-PCI, the trials found no differences in quality of life or in functional status.

## Conclusions

This review provided an overview of patient management following PCI. Follow-up assessments post-PCI generally occur at 1, 6, and 12 months, and, in patients who are well-managed, yearly thereafter. Each follow-up visit should include assessment for appropriate lifestyle modification and risk factor reduction, receipt of appropriate medical therapy for secondary prevention, and symptoms indicative of restenosis. Cardiac rehabilitation should be prescribed at discharge or during the first follow-up visit post-PCI. Noninvasive testing post-PCI should be performed selectively. Routine stress testing is rarely appropriate in asymptomatic patients in the first 2 years following PCI. It can be considered in certain high-risk patient subgroups, such as patients with a history of silent ischemia or high-risk prior PCI, as well as patients needing clearance to return to work. Selection of which noninvasive testing modality to use should depend on the indication for testing, the individual patient profile, and, when used for restenosis detection, the diagnostic performance of the test.

## Funding support and author disclosures

The authors have reported that they have no relationships relevant to the contents of this paper to disclose.

## References

[1] Roy S., Kabach M., Patel D.B., Guzman L.A., Jovin I.S. (2022). Radial artery access complications: prevention, diagnosis and management. Cardiovasc Revascularization Med.

[2] Merriweather N., Sulzbach-Hoke L.M. (2012). Managing risk of complications at femoral vascular access sites in percutaneous coronary intervention. Crit Care Nurse.

[3] Virani S.S., Newby L.K., Arnold S.V. (2023). 2023 AHA/ACC/ACCP/ASPC/NLA/PCNA guideline for the management of patients with chronic coronary disease: a report of the American Heart Association/American College of Cardiology joint committee on clinical practice guidelines. Circulation.

[4] Smith S.C., Benjamin E.J., Bonow R.O. (2011). AHA/ACCF secondary prevention and risk reduction therapy for patients with coronary and other atherosclerotic vascular disease: 2011 update. Circulation.

[5] Suissa K., Larivière J., Eisenberg M.J. (2017). Efficacy and safety of smoking cessation interventions in patients with cardiovascular disease. Circulation: Cardiovascular Quality and Outcomes.

[6] Stead L.F., Koilpillai P., Fanshawe T.R., Lancaster T. (2016). Combined pharmacotherapy and behavioural interventions for smoking cessation. Cochrane Database Syst Rev.

[7] Barua Rajat S., Rigotti Nancy A., Benowitz Neal L. (2018). 2018 ACC Expert Consensus decision Pathway on tobacco cessation treatment. J Am Coll Cardiol.

[8] Lawton J.S., Tamis-Holland Jacqueline E., Bangalore S. (2022). 2021 ACC/AHA/SCAI guideline for coronary artery revascularization. J Am Coll Cardiol.

[9] Levine G.N., Steinke E.E., Bakaeen F.G. (2012). Sexual activity and cardiovascular disease. Circulation.

[10] Vrints C., Andreotti F., Koskinas K.C. (2024). 2024 ESC guidelines for the management of chronic coronary syndromes: developed by the task force for the management of chronic coronary syndromes of the European Society of Cardiology (ESC) Endorsed by the European Association for Cardio-Thoracic Surgery (EACTS). Eur Heart J.

[11] Delgado-Lista J., Alcala-Diaz J.F., Torres-Peña J.D. (2022). Long-term secondary prevention of cardiovascular disease with a Mediterranean diet and a low-fat diet (CORDIOPREV): a randomised controlled trial. Lancet.

[12] Whelton P.K., Carey R.M., Aronow W.S. (2018). 2017 ACC/AHA/AAPA/ABC/ACPM/AGS/APhA/ASH/ASPC/NMA/PCNA guideline for the prevention, detection, evaluation, and management of high blood pressure in adults: a Report of the American College of Cardiology/American heart association task force on clinical practice guidelines. J Am Coll Cardiol.

[13] Filippou C.D., Tsioufis C.P., Thomopoulos C.G. (2020). Dietary approaches to Stop hypertension (DASH) diet and blood pressure reduction in adults with and without hypertension: a systematic review and meta-analysis of randomized controlled trials. Adv Nutr.

[14] Qaseem A., Wilt T.J., Kansagara D., Horwitch C., Barry M.J., Forciea M.A. (2018). Hemoglobin A1c targets for glycemic control with pharmacologic therapy for nonpregnant adults with type 2 diabetes mellitus: a guidance statement update from the American College of physicians. Ann Intern Med.

[15] Sattar N., Lee M.M.Y., Kristensen S.L. (2021). Cardiovascular, mortality, and kidney outcomes with GLP-1 receptor agonists in patients with type 2 diabetes: a systematic review and meta-analysis of randomised trials. Lancet Diabetes Endocrinol.

[16] Grundy S.M., Stone N.J., Bailey A.L. (2019). 2018 AHA/ACC/AACVPR/AAPA/ABC/ACPM/ADA/AGS/APhA/ASPC/NLA/PCNA guideline on the management of blood cholesterol: a report of the American College of Cardiology/American Heart Association task force on clinical practice guidelines. J Am Coll Cardiol.

[17] Lloyd-Jones D.M., Morris Pamela B., Ballantyne C.M. (2022). 2022 ACC expert consensus decision pathway on the role of nonstatin therapies for LDL-cholesterol lowering in the management of atherosclerotic cardiovascular disease risk. J Am Coll Cardiol.

[18] Lincoff A.M., Brown-Frandsen K., Colhoun H.M. (2023). Semaglutide and cardiovascular outcomes in obesity without diabetes. N Engl J Med.

[19] Tardif J.C., Kouz S., Waters D.D. (2019). Efficacy and safety of low-dose colchicine after myocardial infarction. N Engl J Med.

[20] Dalal H.M., Doherty P., Taylor R.S. (2015). Cardiac rehabilitation. BMJ Br Med J (Clin Res Ed).

[21] Buckingham S.A., Taylor R.S., Jolly K. (2016). Home-based versus centre-based cardiac rehabilitation: abridged Cochrane systematic review and meta-analysis. Open Heart.

[22] Dunlay S.M., Pack Q.R., Thomas R.J., Killian J.M., Roger V.L. (2014). Participation in cardiac rehabilitation, readmissions, and death after acute myocardial infarction. Am J Med.

[23] Anderson L., Oldridge N., Thompson D.R. (2016). Exercise-based cardiac rehabilitation for coronary heart disease: cochrane systematic review and meta-analysis. J Am Coll Cardiol.

[24] Cohen B.E., Edmondson D., Kronish I.M. (2015). State of the art review: depression, stress, anxiety, and cardiovascular disease. Am J Hypertens.

[25] Boardman H.M., Hartley L., Eisinga A. (2015). Hormone therapy for preventing cardiovascular disease in post-menopausal women. Cochrane Database Syst Rev.

[26] Cho L., Kaunitz A.M., Faubion S.S. (2023). Rethinking menopausal hormone therapy: for whom, what, when, and how long?. Circulation.

[27] Giacoppo D., Matsuda Y., Fovino L.N. (2021). Short dual antiplatelet therapy followed by P2Y12 inhibitor monotherapy vs. prolonged dual antiplatelet therapy after percutaneous coronary intervention with second-generation drug-eluting stents: a systematic review and meta-analysis of randomized clinical trials. Eur Heart J.

[28] Kang J., Rizas K.D., Park K.W. (2023). Dual antiplatelet therapy de-escalation in acute coronary syndrome: an individual patient meta-analysis. Eur Heart J.

[29] Mehran R., Baber U., Sharma Samin K. (2019). Ticagrelor with or without aspirin in high-risk patients after PCI. N Engl J Med.

[30] Lee S.-Y., Jeong Y.-H., Yun K.H. (2023). P2Y12 inhibitor monotherapy combined with colchicine following PCI in ACS patients. JACC Cardiovasc Interv.

[31] Abraham N.S., Hlatky M.A., Antman E.L. (2010). ACCF/ACG/AHA 2010 expert consensus document on the concomitant use of proton pump inhibitors and thienopyridines: a focused update of the ACCF/ACG/AHA 2008 Expert Consensus document on reducing the gastrointestinal risks of antiplatelet therapy and NSAID use. J Am Coll Cardiol.

[32] Tantry U.S., Kereiakes D.J., Gurbel P.A. (2011). Clopidogrel and proton pump inhibitors. JACC Cardiovasc Interv.

[33] Levine G.N., Bates E.R., Bittl J.A. (2016). 2016 ACC/AHA guideline focused update on duration of dual antiplatelet therapy in patients with coronary artery disease: a report of the American College of Cardiology/American Heart Association task force on clinical practice guidelines: an update of the 2011 ACCF/AHA/SCAI guideline for percutaneous coronary intervention, 2011 ACCF/AHA guideline for coronary artery bypass graft surgery, 2012 ACC/AHA/ACP/AATS/PCNA/SCAI/STS guideline for the diagnosis and management of patients with stable ischemic heart disease, 2013 ACCF/AHA guideline for the management of ST-elevation myocardial infarction, 2014 AHA/ACC guideline for the management of patients with non–ST-elevation acute coronary syndromes, and 2014 ACC/AHA guideline on perioperative cardiovascular evaluation and management of patients undergoing noncardiac surgery. Circulation.

[34] Siller-Matula J.M., Trenk D., Krähenbühl S., Michelson A.D., Delle-Karth G. (2014). Clinical implications of drug–drug interactions with P2Y12 receptor inhibitors. J Thromb Haemostasis.

[35] Lopes R.D., Hong H., Harskamp R.E. (2019). Safety and efficacy of antithrombotic strategies in patients with atrial fibrillation undergoing percutaneous coronary intervention: a network meta-analysis of randomized controlled trials. JAMA Cardiol.

[36] Angiolillo D.J., Bhatt D.L., Cannon C.P. (2021). Antithrombotic therapy in patients with atrial fibrillation treated with oral anticoagulation undergoing percutaneous coronary intervention. Circulation.

[37] Pisters R., Lane D.A., Nieuwlaat R., de Vos C.B., Crijns H.J., Lip G.Y. (2010). A novel user-friendly score (HAS-BLED) to assess 1-year risk of major bleeding in patients with atrial fibrillation: the Euro Heart Survey. Chest.

[38] Naito R., Miyauchi K., Yasuda S. (2022). Rivaroxaban monotherapy vs combination therapy with antiplatelets on total thrombotic and bleeding events in atrial fibrillation with stable coronary artery disease: a post hoc secondary analysis of the AFIRE trial. JAMA Cardiology.

[39] Kumbhani D.J., Cannon C.P., Beavers C.J. (2021). 2020 ACC Expert Consensus decision Pathway for anticoagulant and antiplatelet therapy in patients with atrial fibrillation or venous thromboembolism undergoing percutaneous coronary intervention or with atherosclerotic cardiovascular disease. J Am Coll Cardiol.

[40] Gajanana D., Rogers T., Iantorno M. (2018). Antiplatelet and anticoagulation regimen in patients with mechanical valve undergoing PCI – State-of-the-art review. Int J Cardiol.

[41] Giustino G., Colombo A., Camaj A. (2022). Coronary in-stent restenosis: JACC state-of-the-art review. J Am Coll Cardiol.

[42] Madhavan M.V., Kirtane A.J., Redfors B. (2020). Stent-related adverse events >1 Year after percutaneous coronary intervention. J Am Coll Cardiol.

[43] Sakamoto A., Sato Y., Kawakami R. (2021). Risk prediction of in-stent restenosis among patients with coronary drug-eluting stents: current clinical approaches and challenges. Expet Rev Cardiovasc Ther.

[44] Kawai K., Virmani R., Finn A.V. (2022). In-stent restenosis. Interventional Cardiology Clinics.

[45] Cassese S., Byrne R.A., Tada T. (2014). Incidence and predictors of restenosis after coronary stenting in 10 004 patients with surveillance angiography. Heart.

[46] Magalhaes M.A., Minha Sa, Chen F. (2014). Clinical presentation and outcomes of coronary in-stent restenosis across 3-stent generations. Circulation: Cardiovascular Interventions.

[47] Moussa I.D., Mohananey D., Saucedo J. (2020). Trends and outcomes of restenosis after coronary stent implantation in the United States. J Am Coll Cardiol.

[48] Ruygrok P.N., Webster M.W.I., de Valk V. (2001). Clinical and angiographic factors associated with asymptomatic restenosis after percutaneous coronary intervention. Circulation.

[49] Cassese S., Byrne R.A., Schulz S. (2015). Prognostic role of restenosis in 10 004 patients undergoing routine control angiography after coronary stenting. Eur Heart J.

[50] Hannan E.L., Samadashvili Z., Cozzens K. (2014). Appropriateness of diagnostic catheterization for suspected coronary artery disease in New York state. Circulation: Cardiovascular Interventions.

[51] Winchester D.E., Maron D.J., Blankstein R. (2023). ACC/AHA/ASE/ASNC/ASPC/HFSA/HRS/SCAI/SCCT/SCMR/STS 2023 multimodality appropriate use Criteria for the detection and risk assessment of chronic coronary disease. J Am Coll Cardiol.

[52] Shiomi H., Morimoto T., Kitaguchi S. (2017). The ReACT trial: randomized evaluation of routine follow-up coronary angiography after percutaneous coronary intervention trial. JACC Cardiovasc Interv.

[53] Garzon P.P., Eisenberg M.J. (2001). Functional testing for the detection of restenosis after percutaneous transluminal coronary angioplasty: a meta-analysis. Can J Cardiol.

[54] Neumann F.-J., Sousa-Uva M., Ahlsson A. (2019). 2018 ESC/EACTS Guidelines on myocardial revascularization. Eur Heart J.

[55] Ghotbi A.A., Kjaer A., Hasbak P. (2014). Review: comparison of PET rubidium-82 with conventional SPECT myocardial perfusion imaging. Clin Physiol Funct Imaging.

[56] Mc Ardle B.A., Dowsley T.F., deKemp R.A., Wells G.A., Beanlands R.S. (2012). Does rubidium-82 PET have superior accuracy to SPECT perfusion imaging for the diagnosis of obstructive coronary disease?: a systematic review and meta-analysis. J Am Coll Cardiol.

[57] Bernhardt P., Spiess J., Levenson B. (2009). Combined assessment of myocardial perfusion and late gadolinium enhancement in patients after percutaneous coronary intervention or bypass grafts: a multicenter study of an integrated cardiovascular magnetic resonance protocol. JACC Cardiovasc Imaging.

[58] Symons R., Zimmerman Stefan L., Bluemke David A. (2019). CMR and CT of the patient with cardiac devices. JACC (J Am Coll Cardiol): Cardiovascular Imaging.

[59] Narula J., Chandrashekhar Y., Ahmadi A. (2021). SCCT 2021 Expert Consensus document on coronary computed tomographic angiography: a Report of the society of cardiovascular computed tomography. J Cardiovasc Comput Tomogr.

[60] Taylor Allen J., Cerqueira M., Hodgson J.M. (2010). ACCF/SCCT/ACR/AHA/ASE/ASNC/NASCI/SCAI/SCMR 2010 appropriate use Criteria for cardiac computed tomography. J Am Coll Cardiol.

[61] Tang C.X., Guo B.J., Schoepf J.U. (2021). Feasibility and prognostic role of machine learning-based FFR(CT) in patients with stent implantation. Eur Radiol.

[62] Andreini D., Mushtaq S., Pontone G. (2020). CT perfusion versus coronary CT angiography in patients with suspected in-stent restenosis or CAD progression. JACC Cardiovasc Imaging.

[63] Park D.W., Kang D.Y., Ahn J.M. (2022). Routine functional testing or standard care in high-risk patients after PCI. N Engl J Med.

[64] Kim H., Kang D.Y., Lee J. (2023). Routine stress testing in diabetic patients after percutaneous coronary intervention: the POST-PCI trial. Eur Heart J.

[65] Lee J., Kang D.-Y., Kim H. (2024). Routine stress testing after PCI in patients with and without acute coronary syndrome: a secondary analysis of the post-PCI randomized clinical trial. JAMA Cardiology.

[66] Eisenberg M.J., Blankenship J.C., Huynh T. (2004). Evaluation of routine functional testing after percutaneous coronary intervention. Am J Cardiol.

[67] Eisenberg M.J., Wilson B., Lauzon C. (2007). Routine functional testing after percutaneous coronary intervention: results of the aggressive diagnosis of restenosis in high-risk patients (ADORE II) trial. Acta Cardiol.

